# MicroRNA-205-5p targets the HOXD9-Snail1 axis to inhibit triple negative breast cancer cell proliferation and chemoresistance

**DOI:** 10.18632/aging.202363

**Published:** 2021-01-10

**Authors:** Li-Fen Lin, Yi-Ting Li, Hui Han, Shun-Guo Lin

**Affiliations:** 1 Department of Breast Surgery, Fujian Medical University Union Hospital, Fuzhou, Fujian 350001, P.R. China

**Keywords:** microRNA, breast cancer, chemotherapy resistance, EMT

## Abstract

MicroRNA-205 (miR-205) is believed to be related to the progress of tumors. HOXD9 has been proved to be expressed abnormally in several kinds of cancers. However, the role of miR-205 and HOXD9 in breast cancer remains unclear. The biological role of miR-205 in breast cancer cell proliferation and chemoresistance was investigated. The expression of miR-205 in clinical tissues and breast cancer cell lines were analyzed using quantitative real-time PCR test (qRT-PCR). Overexpression and knockdown models of miR-205 were established to study cell proliferation and chemotherapy-resistant. Moreover, the potential relationships between miR-205 and HOXD9/Snail1 were measured using qRT-PCR, western blot, and chemotherapy-resistant study. miR-205 was lowly expressed in breast cancer tissues and cell lines. Overexpression of miR-205 could inhibit cell proliferation and chemotherapy-resistance. Moreover, we proved that miR-205 could target the HOXD9-Snail1 axis to suppress triple negative breast cancer cell proliferation and chemoresistance. The activation of Snail1 gene by HOXD9 was also proved in this study. The present study may provide a novel insight for the therapeutic strategies of breast cancer through targeting miR-205/HOXD9/Snail1.

## INTRODUCTION

Breast cancer is believed to be one of the most common malignant cancer among women, and it is characterized by high morbidity and mortality [1]. Clinically, breast cancer can be divided into five subtypes based on the specific marker level on the surface of breast cancer cells [2]. Triple-negative breast cancer (TNBC) accounts for 15% of various subtypes [3], and TNBC is considered the most dangerous type of breast cancer. The disease is mainly diagnosed in young women [4]. This disease has various clinical features, including high malignancy, strong invasiveness, easy recurrence, easy metastasis, and poor prognosis [5, 6]. Chemotherapy drugs such as Epirubicin and Paclitaxel are commonly used for TNBC treatment [7]. However, more than 50% of patients experience tumor recurrence within 3 to 5 years after treatment. Acquired chemotherapy resistance could lead to secondary recurrence or metastasis of the tumor [8]. Therefore, unfolding the resistance mechanism of TNBC and intervene strategies to the chemotherapy resistance of TNBC present great clinical significance.

MicroRNA (miRNA) is a type of non-coding RNA, and miRNAs can degrade the mRNA or participate in post-transcriptional regulation, thereby inhibiting the target gene expression [9]. MicroRNA-205 (miR-205) is located in the second intron in the LOC642587 locus of human chromosome 1 (1q32.2) and is 110 bases in length. Several experiments have proved that miR-205 was linked with the progression of various malignant tumors in humans through affecting the proliferation, differentiation, invasion, and apoptosis of tumor cells. In prostate cancer, overexpression of miR-205 can inhibit cell invasion and metastasis through epithelial-mesenchymal transition (EMT) [10]. However, the level of miR-205 is increased in ovarian cancer and lung cancer [11, 12]. The level of miR-205 was increased in lung squamous cell carcinoma [13], neck squamous cell carcinoma [14], and endometrial carcinoma [15]. On the contrary, the expressions of miR-205 were decreased in bladder cancer [16]. The specific role of miR-205 in TNBC proliferation and chemotherapy resistance is not clear.

The HOXD 9 gene is a member of the HOXD family, which has been believed to be closely linked with progression of tumor. For example, HOXD10 is abnormally expressed in several types of cancers, cervical cancer [17], ovarian cancer [18], endometrial cancer [19], lung cancer [20], and leukemia [21]. Meanwhile, HOXD10 can induce the level of P53 and suppress the level of oncogene Snail1 in breast cancer [22]. Meanwhile, HOXD9 could be regulated by miR-126 [23], and miR-10b can interact with HOXD10 to promote breast cancer metastasis [24]. However, whether miR-205 could affect HOXD9 expression and further influence breast cancer cells viability remain unknown.

In this study, we measured the level of miR-205 in cells and tissues. The function of miR-205 in cell lines was investigated. Meanwhie, the specific mechanism of miR-205 and Snail1/HOXA9 was investigated. The present study may provide a novel thought for the treatment of breast cancer by targeting miR-205/HOXD9/Snail1.

## RESULTS

### miR-205 was low expressed in TNBC tissue

**Figure 1:**
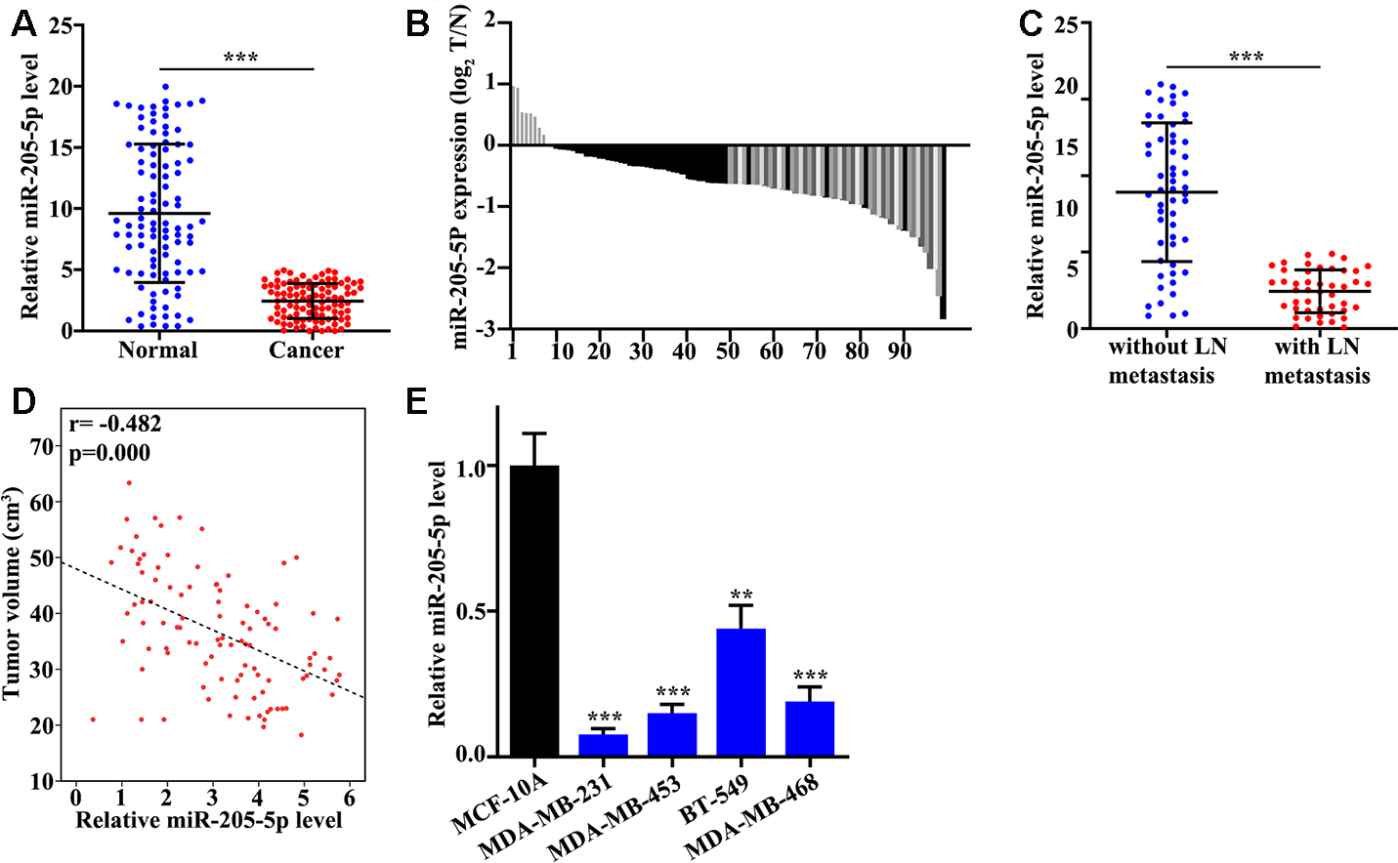
**miR-205 is low expressed in TNBC tissues.** (**A**) mRNA abundance analysis of miR-205 in 100 TNBC tissues and paired normal tissues. (**B**) The sample distribution analysis of the low expression in tumor tissue and adjacent tissues among 100 pairs of specimens. (**C**) Detection of miR-205 expression in TNBC tissues with or without lymph node metastasis. (**D**) Relationship analysis between miR-205 expression and tumor volume. (**E**) qRT-PCR analysis of the miR-205 abundance in breast cancer cell lines and normal breast cell lines. **P<0.05, ***P<0.001.

In this study, we found that miR-205 in TNBC tissue was remarkably lower than control (P<0.001) (Figure 1A). Meanwhile, the distribution of low miR-205 expression was analyzed indicating that 92% (92 of 100) low miR-205 expression can be measured in TNBC tissues. Moreover, miR-205 expression in TNBC tissues with lymph node metastasis was significantly down-regulated (Figure 1C). Correlation analysis of miR-205 expression and tumor volume suggested that miR-205 level was negatively linked to the tumor volume (P=0.000) (Figure 1D). The level of miR-205 in BT-549, MDA-MB-468, MDA-MB-453, MDA-MB-231, and MCF-10A was measured. Significant lower expression of miR-205 in MDA-MB-231 and remarkable higher level of miR-205 in BT-549 were observed (Figure 1E). Therefore, MDA-MB-231 and BT-549 cells were used for further experiments.

### miR-205 inhibited TNBC cell proliferation

**Figure 2:**
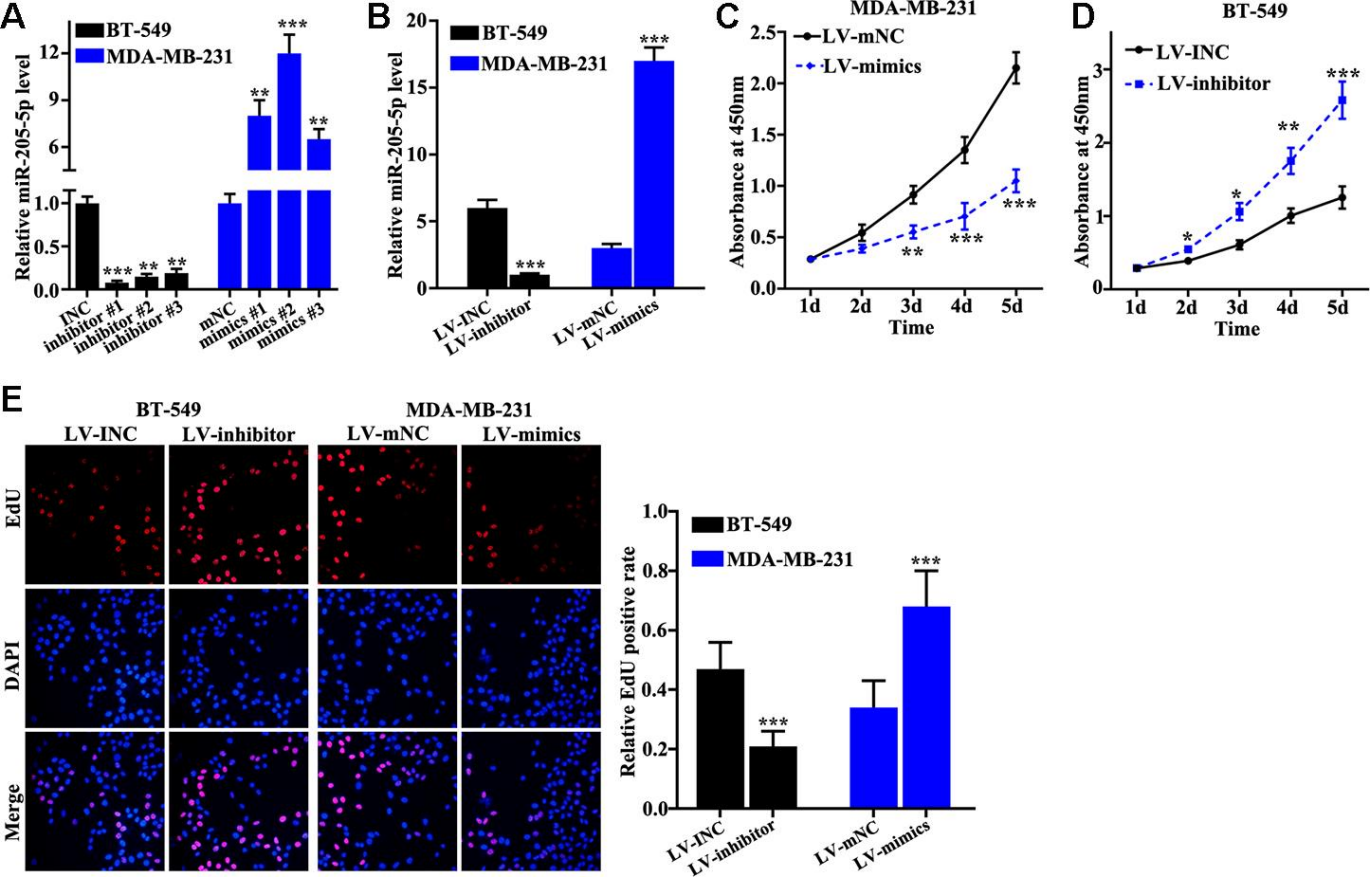
**miR-205 inhibits breast cancer cell proliferation.** (**A**) qRT-PCR was used to investigate knockdown efficiency and over-expression efficiency. (**B**) qRT-PCR was used to identify the effects of miR-205 knockdown treatment in BT-549 cells and miR-205 overexpression treatment in MDA-MB-231 cells. (**C**) miR-205 overexpression in MDA-MB-231 cells significantly promoted cell proliferation. (**D**) miR-205 knockdown in BT-549 cells significantly inhibited cell proliferation. (**E**) edu experiment was employed to study the cell proliferation of miR-205 knockdown treatment in BT-549 cells and miR-205 overexpression treatment in MDA-MB-231 cells. *P<0.05, **P<0.01,***P<0.001.

Overexpression and knockdown of miR-205 models in MDA-MB-231 and BT-549 cells were successfully established. Inhibitor #1 and mimics #2 were used (Figure 2A), and the efficiency of miR-205 overexpression and knockdown models in BT-549 and MDA-MB-231 cells was measured (Figure 2B). miR-205 overexpression in MDA-MB-231 cells can remarkably promote cell proliferation compared with normal MDA-MB-231 cells on days 3, 4, 5 (Figure 2C). However, miR-205 knockdown in BT-549 cells can markedly inhibited cell proliferation compared with normal BT-549 cells on days 3, 4, 5 (Figure 2D). Edu experiment was applied to detect cell number changes in miR-205 knockdown treated BT-549 cells and miR-205 overexpression treated MDA-MB-231 cells. miR-205 knockdown treated BT-549 cells can remarkably inhibit cell proliferation. Meanwhile, miR-205 overexpression treated MDA-MB-231 cells (Figure 2E) can markedly promote cell proliferation *in vitro*.

### miR-205 inhibited chemotherapy resistance of TNBC cells

**Figure 3:**
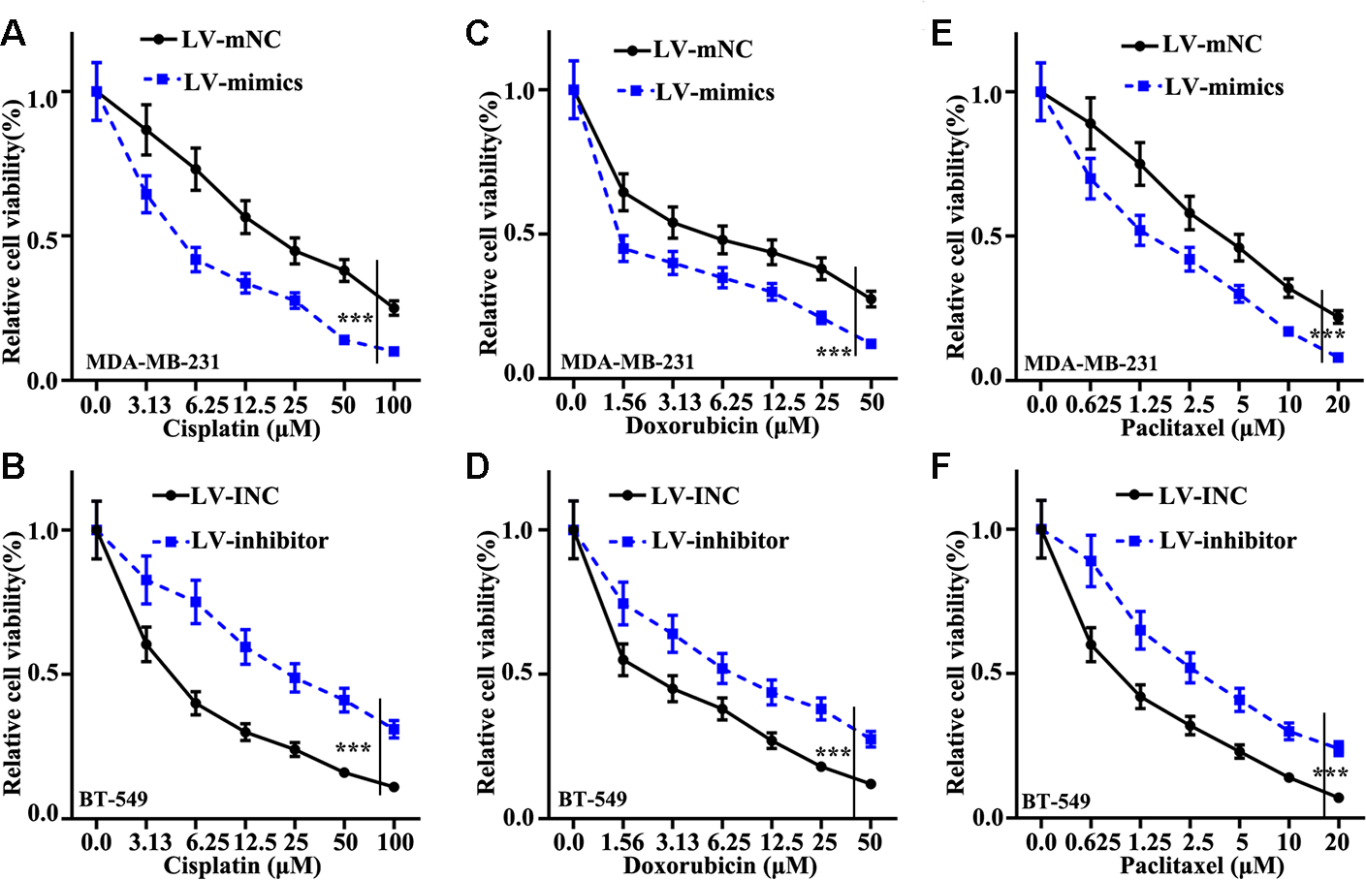
**Effect of miR-205 on chemotherapy sensitivity of TNBC cells.** (**A**) Cell proliferation analysis of Cisplatin in miR-205 overexpression treatment in MDA-MB-231 cells. (**B**) Cell proliferation analysis of Cisplatin in miR-205 knockdown treatment in BT-549 cells. (**C**) Cell proliferation analysis of Doxorubicin in miR-205 overexpression treatment in MDA-MB-231 cells. (**D**) Cell proliferation analysis of Doxorubicin in miR-205 knockdown treatment in BT-549 cells. (**E**) Cell proliferation analysis of Paclitaxel in miR-205 overexpression treatment in MDA-MB-231 cells. (**F**) Cell proliferation analysis of Paclitaxel in miR-205 knockdown treatment in BT-549 cells. ***P<0.001.

Cisplatin, doxorubicin, and paclitaxel were applied to measure the influence of miR-205 on chemotherapy sensitivity of TNBC. We found that miR-205 overexpression treated MDA-MB-231 cells can remarkably suppress cell viability (Figure 3A, 3C, 3E). However, miR-205 knockdown treated BT-549 cells can markedly promote cell viability compared normal BT-549 cells (Figure 3B, 3D, 3F). These results revealed that miR-205 knockdown treated BT-549 cells and miR-205 overexpression treated MDA-MB-231 cells was closely linked with the chemotherapy resistance of TNBC cells.

### miR-205 regulated Snail1 expression mediating TNBC proliferation and chemotherapy resistance

**Figure 4:**
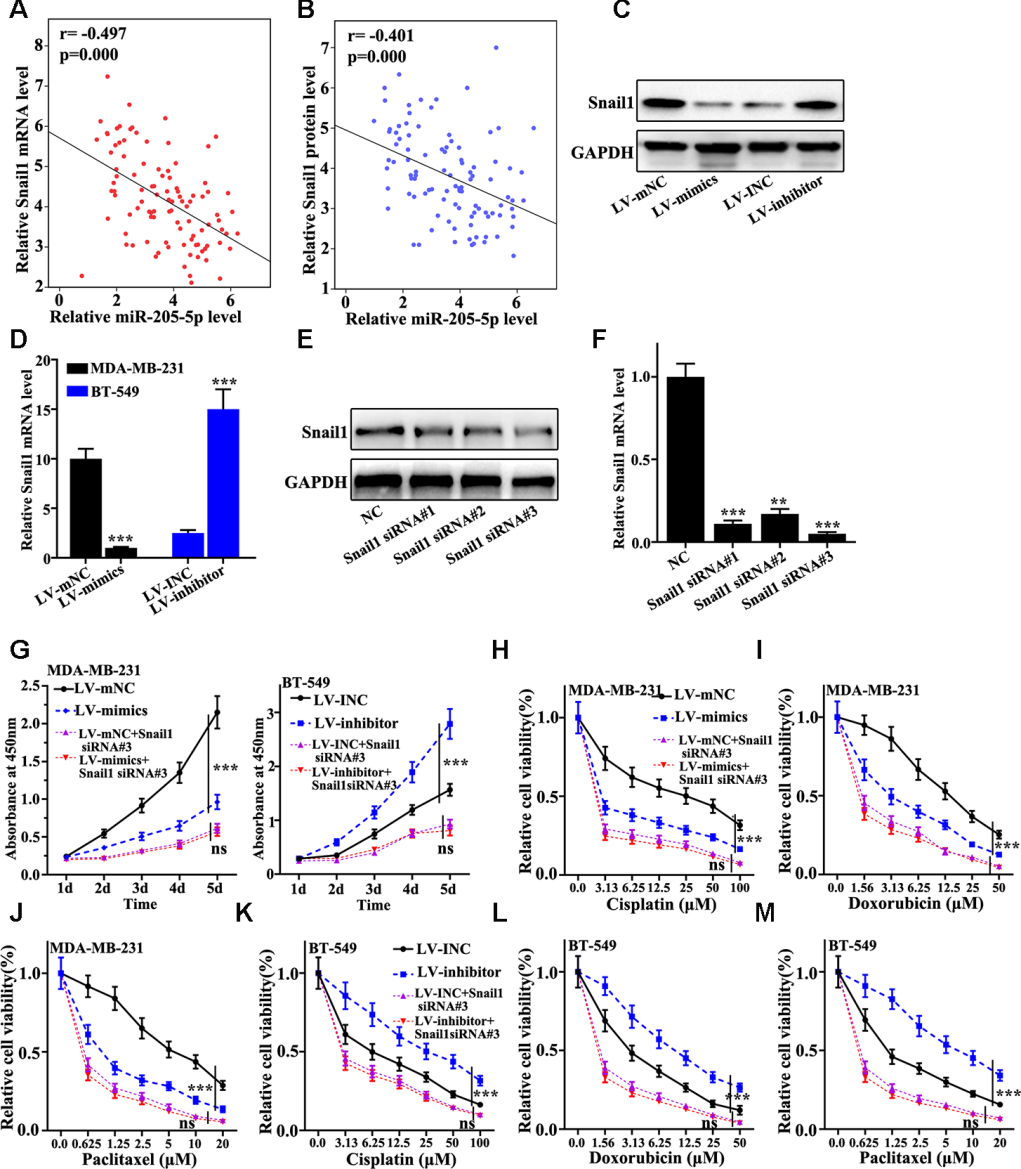
**miR-205 regulates Snail1 expression to mediate breast cancer cell proliferation and chemotherapy resistance.** (**A**) Verifying the correlation between miR-205 and Snail1 mRNA in 100 TNBC specimens. (**B**) The correlation between miR-205 and Snail1 protein expression was calculated in 100 TNBC specimens. (**C**) Western blot analysis of the Snail1 protein and mRNA expression in miR-205 knockdown treatment in BT-549 cells and miR-205 overexpression treatment in MDA-MB-231 cells. (**D**) qRT-PCR analysis of the Snail1 protein and mRNA expression in miR-205 knockdown treatment in BT-549 cells and miR-205 overexpression treatment in MDA-MB-231 cells. (**E**) Western blot are used to screen the Snail1 siRNA sequences with the best interference effect for subsequent experiments. (**F**) qRT-PCR are used to screen the Snail1 siRNA sequences with the best interference effect for subsequent experiments. (**G**) CCK8 was used to analyze the Snail1 knockdown effect with the miR-205 knockdown treated BT-549 cells and miR-205 overexpression treated MDA-MB-231 cells. (**H**) Cell proliferation analysis of the miR-205 overexpression and Snail1 knockdown treated MDA-MB-231 cells with Cisplatin treatment. (**I**) Cell proliferation analysis of the miR-205 overexpression and Snail1 knockdown treated MDA-MB-231 cells with Doxorubicin treatment. (**J**) Cell proliferation analysis of the miR-205 overexpression and Snail1 knockdown treated MDA-MB-231 cells with three Paclitaxel treatment. (**K**) Cell proliferation analysis of the miR-205 and Snail1 knockdown treated BT-549 cells with Cisplatin treatment. (**L**) Cell proliferation analysis of the miR-205 and Snail1 knockdown treated BT-549 cells with Doxorubicin treatment. (**M**) Cell proliferation analysis of the miR-205 and Snail1 knockdown treated BT-549 cells with Paclitaxel treatment. ***P<0.001.

The relationship between the mRNA abundance of miR-205 and Snail1 was further investigated. We found that negative correlation between the expression of Snail1 and the mRNA level of miR-205 could be observed (Figure 4A and 4B). Figure 4C indicated that Snail1 protein expression in miR-205 overexpression treated MDA-MB-231 cells was significant lower than that in normal MDA-MB-231 cells. However, Snail1 protein expression in miR-205 knockdown treated BT-549 cells were remarkably higher than normal BT-549 (Figure 4C). Similar results of Snail1 mRNA level could be observed in miR-205 knockdown treated BT-549 cells and miR-205 overexpression treated MDA-MB-231 cells (Figure 4D). These results indicated that miR-205 could influence the Snail1 expression. Moreover, we designed three siRNAs to knockdown the Snail1 gene in MDA-MB-231 cells. siRNA 3# was an effective sequence to knockdown Snail1 gene. Therefore, siRNA 3# treated MDA-MB-231 cells was selected for further study (Figure 4E and 4F). Figure 4G showed that miR-205 overexpression and Snail1 knockdown treatment could significantly inhibit cell proliferation (P<0.001). Meanwhile, miR-205 and Snail1 knockdown treatment could significantly promote cell proliferation (P<0.001). Moreover, Figure 4H, Figure 4I, 4J showed the cell viability of miR-205 overexpression and Snail1 knockdown treated MDA-MB-231 cells. The results suggested that Snail1 knockdown treated MDA-MB-231 cells could markedly suppress the cell viability compared with that in normal miR-205 overexpression MDA-MB-231 (P<0.05). Similar results were harvested in miR-205 and Snail1 knockdown treated BT-549 cells (P<0.001) (Figure 4K, 4L, 4M). These results revealed that Snail1 knockdown in miR-205 knockdown treated BT-549 cells and miR-205 overexpression treated MDA-MB-231 cells was linked with cell viability changes induced by drug treatments.

### miR-205 targeted regulation of HOXD9 gene

**Figure 5:**
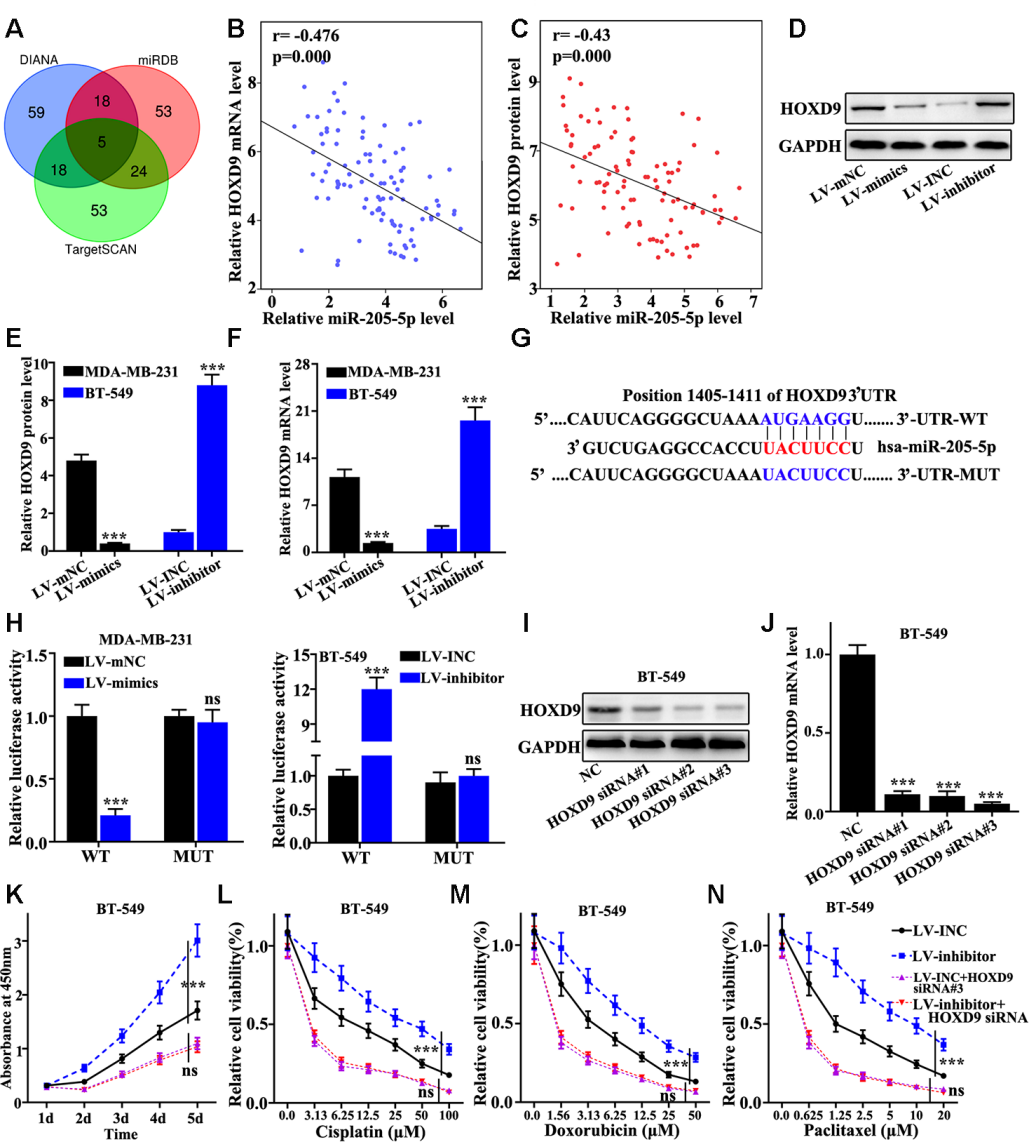
**miR-205 targeted regulation of HOXD9.** (**A**) Three major databases were used to predict target genes of miR-205. Based on the final biological function, HOXD9 was selected. (**B**) The correlation between miR-205 and HOXD9 mRNA expression was calculated in 100 pairs of TNBC specimens. (**C**) The correlation between miR-205 and HOXD9 protein expression was calculated in 100 pairs of TNBC specimens. (**D**) HOXD9 protein expression in miR-205 overexpression treated MDA-MB-231 cells was lower than that in normal MDA-MB-231 cells. (**E**) Western blot analysis of the HOXD9 protein and mRNA expression in miR-205 knockdown treated BT-549 cells and miR-205 overexpression treated MDA-MB-231 cells. (**F**) qRT-PCR analysis of the HOXD9 protein and mRNA expression in miR-205 knockdown treated BT-549 cells and miR-205 overexpression treated MDA-MB-231 cells. (**G**) Construction of HOXD9 mRNA 3’UTR wild-type and mutant plasmids using potential binding sites of miR-205 and HOXD9 mRNA. (**H**) Dual luciferase experiment was used to verify the binding of miR-205 and HOXD9 mRNA. (**I**) Western blot analysis of HOXD9 siRNA. (**J**) qRT-PCR analysis of HOXD9 siRNA. (**K**) Cell proliferation analysis of the miR-205 and HOXD9 knockdown treated BT-549 cells after different incubation time. (**L**) Cell proliferation analysis of the miR-205 and HOXD9 knockdown treated BT-549 cells with Cisplatin treatment. (**M**) Cell proliferation analysis of the miR-205 and HOXD9 knockdown treated BT-549 cells with Doxorubicin treatment. (**N**) Cell proliferation analysis of the miR-205 and HOXD9 knockdown treated BT-549 cells with Paclitaxel treatment. ***P<0.001.

Targetscan, miRDB, and DIANA were screened to obtain the potential target genes of miR-205. The results showed that five genes were included in the overlap of three databases. Based on the biological function, HOXD9 gene was selected for further analysis (Figure 5A). Negative correlation between the mRNA expression of miR-205 and the level of HOXD9 was observed (Figure 5B and 5C). Figure 5D suggested that HOXD9 protein level in miR-205 overexpression treated MDA-MB-231 cells was significantly lower than that in normal MDA-MB-231 cells. However, HOXD9 protein expression in miR-205 knockdown treated BT-549 cells were remarkably higher compared with that in normal BT-549 (Figure 5D). Similar results of Snail1 mRNA level could be observed in miR-205 knockdown treated BT-549 cells and miR-205 overexpression treated MDA-MB-231 cells (Figure 5E and 5F). Moreover, we constructed HOXD9 mRNA 3 UTR wild-type and mutant plasmids (Figure 5G). Dual luciferase experiment identified that miR-205 could bind to the mRNA 3 UTR wild-type of the HOXD9 gene but not the HOXD9 mutant plasmids (Figure 5H). Furthermore, we designed three siRNAs to knockdown the HOXD9 gene in BT549 cells. siRNA 3# was an effective sequence to knockdown Snail1 gene. Therefore, siRNA 3# treated BT549 cells was selected for further study (Figure 5I and 5J). Cisplatin, doxorubicin, and paclitaxel were used to probe the cell proliferation of the miR-205 knockdown treated BT-549 cells with HOXD9 knockdown treatment. Figure 5K suggested that HOXD9 knockdown could inhibit cell proliferation of the miR-205 knockdown treated BT-549 cells (P<0.001). In addition, Figure 5L, 5M, and 5N showed the cell viability of miR-205 and HOXD9 knockdown treated BT-549 cells. HOXD9 knockdown treated BT-549 cells can remarkably inhibit cell viability after drug treatments compared with control. Therefore, HOXD9 knockdown in miR-205 knockdown treated BT-549 cells was linked with the changes in cell viability induced by drug.

### HOXD9 gene transcriptional activation Snail1 gene

**Figure 6:**
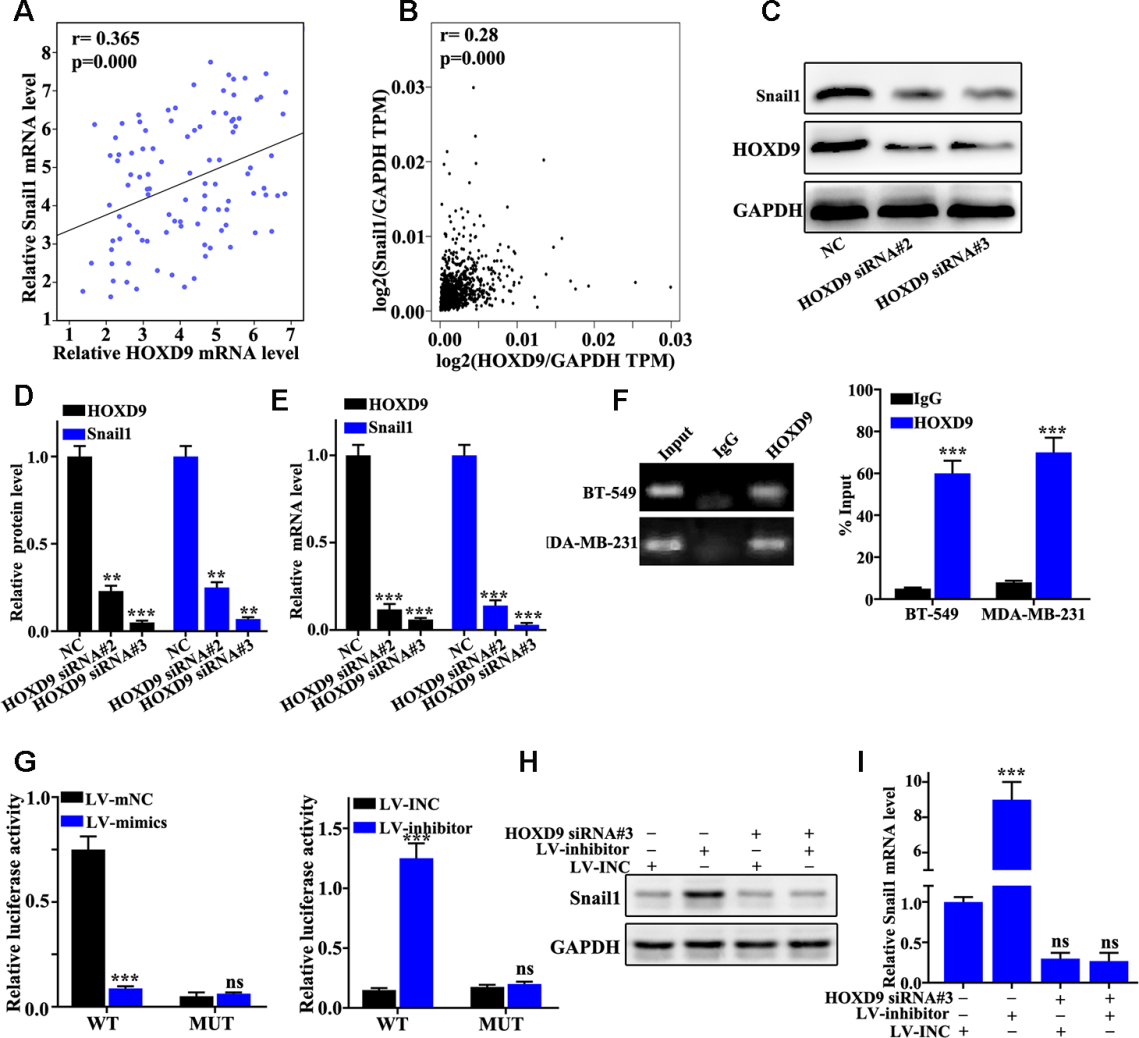
**HOXD9 transcription activates Snail1 expression.** (**A**) Verifying the correlation between HOXD9 and Snail1 mRNA in 100 TNBC specimens. (**B**) The correlation between HOXD9 and Snail1 mRNA expression was calculated in GEPIA database. (**C**) HOXD9 knockdown treatment could effectively inhibit Snail1 protein expression in BT549 cells. (**D**) Western blot analysis of the Snail1 protein expression in HOXD9 knockdown treated BT549 cells. (**E**) qRT-PCR analysis of the Snail1 mRNA expression in HOXD9 knockdown treated BT549 cells. (**F**) ChIP experiments was used to verify the binding site between HOXD9 and the promoter region of Snail1 gene. (**G**) Dual luciferase experiment was employed to verify the binding site between HOXD9 and the wild-type promoter region of Snail1. (**H**) Western blot analysis of the Snail1 protein expression in miR-205 knockdown treatment in BT-549 cells and miR-205 overexpression treatment in MDA-MB-231 cells. (**I**) qRT-PCR analysis of the Snail1 mRNA expression in miR-205 knockdown treatment in BT-549 cells and miR-205 overexpression treatment in MDA-MB-231 cells. **P<0.01,***P<0.001.

We further studied the relationships between the abundance of HOXD9 mRNA and Snail1 with clinical samples and GEPIA online database (Figure 6A and 6B). The results revealed that mRNA abundance of HOXD9 was positively related to the mRNA abundance of Snail1. Figure 6C–6E suggested that HOXD9 knockdown treatment could effectively inhibit Snail1 protein and mRNA expression in BT549 cells (P<0.01). Moreover, ChiP experiments identified that HOXD9 can bind to the promoter region of Snail1 (Figure 6F). Dual luciferase experiment indicated that HOXD9 can bind to the promoter region of the Snail1 mRNA wild-type but not the mutant plasmid (Figure 6G). The Snail1 expression in HOXD9 and miR-205 knockdown treated BT-549 cells were measured (Figure 6H–6I). Snail1 expression could be affected by HOXD9 and miR-205. HOXD9 gene could directly transcriptional active Snail1 gene.

## DISCUSSION

Breast cancer is one of the most common cancers in women, and its incidence is increasing year by year worldwide [25]. As the primary stage of metastasis, EMT is linked with reduction of E-cad expression [26]. The expression of E-cad is modulated by various genes such as NF-κB, MMP family [27], which are affected by miRNA. miRNAs act a key role in promoting or suppressing in breast cancer. For example, the overexpression of miR-21 in MCF7 cells will promote growth and invasion ability of cells [28]; miR-200 family can target ZEB1 and ZEB2 genes to inhibit breast cancer cells [29]. miR-10b is significantly increased in aggressive breast cancer cells [30]. Moreover, the inhibition of miR-205 by TGF-β can promote the occurrence of EMT [31].

It was reported that miR-205 can affect the progression of tumors. However, the function of miR-205 in breast cancer is not well known. The up-regulated miR-205 can inhibit the expression of PTEN to eliminate cell contact inhibition and promote colony formation of tumor cells. Moreover, the up-regulated miR-205 can also target ZEB1 to inhibit breast cancer cell invasion [32]. However, restoration of miR-205 expression in breast cancer can act on Eerb B3 and VEGF-A genes to inhibit the proliferation and invasion of breast cancer cells [33]. This results was consistent to our work. In this study, our evidences suggested that the miR-205 level change was linked with the cell proliferation and chemoresistance of TNBC. miR-205 might be a potential target for TNBC treatment.

Snail1 is a zinc finger transcription factor discovered in recent years. It is a key regulator of tumor cells during EMT [34]. Snail1 acts a key role in tumor cell infiltration and migration [35]. It was reported that Snail1 can be used as an independent negative diagnostic indicator for breast cancer [36], which are closely related to the poor breast cancer differentiation, strong invasiveness, easy metastasis, and short survival time. Moreover, the high expression of Snail1 and Slug in breast invasive ductal carcinoma is closely related to lymph node metastasis. This study shows that the level of Snail1 in TNBC is closely linked with miR-205 in TNBC. Meanwhile, Snail1 and miR-205 play an important role in chemotherapy resistance and cell proliferation. Therefore, miR-205 could regulate the level of Snail1, and further affect chemotherapy resistance and cell proliferation of TNBC cells.

HOXD gene, a special kind of transcriptional regulator, acts a key regulatory role in the process of cell differentiation, proliferation and apoptosis, and the occurrence of various tumors [37]. Petr Novak et al. suggested that HOXD1-HOXD10 genes were down-regulated in breast invasive ductal carcinoma. Reynolds et al [38] revealed that the lack of HOXD9 gene expression in breast cancer can cause p16 expression disorder. p16 protein is a recognized negative regulator of cell cycle and is an important factor in inhibiting cell proliferation and tumorigenesis [39]. HOXD9 level was markedly influenced in Snail1 and miR-205 overexpression and knockdown treated cells. HOXD9 could interact with Snail1 in cellular. Moreover, HOXD9 can regulate Snail1 protein level. Therefore, our results revealed that miR-205 can target the HOXD9-Snail1 to suppress triple negative breast cancer cell proliferation and chemoresistance.

In summary, low miR-205 expression can be observed in TNBC cells and tissues. miR-205 could function the HOXD9-Snail1 to suppress triple negative breast cancer cell proliferation and chemoresistance. This study may provide a novel insight for the treatment of breast cancer through targeting miR-205/HOXD9/Snail1.

## MATERIALS AND METHODS

### Tissues and cell lines

100 TNBC samples and normal tissues were collected in the Fujian Medical University Union Hospital from Jan 2019 to Dec 2019. The research was approved by the ethics committee of Fujian Medical University Union Hospital. The informed consents have been signed by patients. BT-549, MDA-MB-468, MDA-MB-453, MDA-MB-231, and MCF-10A cell lines were obtained from ATCC (Virginia, USA). Cells were cultivated using RPMI 1640 containing 10% FBS (Invitrogen, CA) on the condition of 5% CO_2_ and 37° C. Cells (5×10^5^ each well) were seeded on six-well plate. Lipofectamine 2000 reagent (Thermo Fisher, USA) and OPTI-MEM serum-free medium (M5650, Sigma) were used for cell transfection. 100 nM siRNA was used to construct knockdown model of miR-205. Meanwhile, MiR-205 overexpression model was estabilished using pEZ-Lv201 Vector (Biovector, China). Titer Boost^TM^ and Endo Fectin-Lenti^TM^ (CWBio, China) were applied to co-transfect MDA-MB-231 cells. After 48 h transfection, the supernatant was collected.

### qRT-PCR analysis

CFX96 system (Bio-Rad, USA) was used in this study for qRT-PCR detection. RNA was extracted using M5 SuperPure Extraction Reagent (mei5bio, China). Q225 system (Kubotechnology, China) was used to measure mRNA level. The PCR reaction system included 6μL H_2_O, 2μL cDNA template (10ng), 1μL anti-sense primer (10 nM), 1μL sense primer (10 nM), and 10μL GoldStar Probe Mixture (Low ROX) (CWBio, China). The program qRT-PCR was set as follows: 95° C, 30 seconds, 40 cycles (95° C, 5 seconds, and 60° C, 10 seconds). 2^-ΔΔCt^ cycle method was applied to analyze data. GAPDH was used as internal control. Primers were listed as follows: HOXD9 (Forward: CCGAAACTTCTCAACAACAAG, Reverse: GCCAGAAAGGCACCTGATAGC); miR-205 (Forward: GATGAGCTCAACTGAAGTGGCTAAAGAG, Reverse: GATACGCGTTGAAGTTCTGCCTAATCTA); U6 (Forward: AAAGCAAATCATCGGACGACC, Reverse: GTACAACACATTGTTTCCTCGGA); Snail1 (Forward: TCGGAAGCCTAACTACAGCGA, Reverse: AGATGAGCATTGGCAGCGAG); GAPDH (Forward: AGAAGGCTGGGGCTCATTTG, Reverse: AGGGGCCATCCACAGTCTTC).

### CCK8

100 μL Cells (2×10^3^ cells/well) were plated into 96-well plates, and incubated on the condition of 5% CO_2_, 37° C. After different treatments for 24 h, CCK-8 reagent (Beyotime, China) was added, and OD at 450 nm was measured. The experiment was repeated 3 times with 5 replicate wells in each group.

### Immunofluorescence analysis

Cells were plated into 24-well plates for 48 h. After washing using PBS, cells were fixed using 4% paraformaldehyde for 30 min. Then, cells were washed three times using PBS. Cells were treated with 0.1% Triton for 10 min, and washed with PBS for 5 min. Cells were blocked with goat serum for 1 h, and then incubated using first antibody overnight. After washing 3 times with PBS, cells were incubated with secondary antibody for 1 h. PBS was used to wash cells, and then cells were observed using an inverted fluorescence microscope (IX71, Olympus, Japan).

### Western blot analysis

Protein in cells were lysed with lysis buffer (Beyotime, China) and extracted. Same amount of protein was loaded for 10% SDS-PAGE. Gels were transferred onto a polyvinylidene difluoride layer (Novus, USA). 5% non-fat milk was applied for blocking. After 2 h, membrane was cultivated with anti-Rabbit Snail1(1:1000) (#3879, CST, USA), HOXD9 (1:1000)(#55962, CST, USA) and GAPDH (1:1000) (#2118, CST, USA) overnight.. After washing twice, secondary antibodies were used for incubation for 1 h. After washing twice with TBST buffer, ECL Chemiluminescence Detection Kit (PromoCell, German) was used to treat cells, and Image J software was used to analyze protein bands.

### Detection of double luciferase reporter gene

After plasmid transfection, 100 μL cell lysate was added, and cells were shaken slowly at room temperature for 15 min. 50 μL luciferase detection reagent II was added to 10 μL of cell lysate. Fluorescent luminometer was used to measure the firefly luciferase activity. Then, 50 μL Renilla luciferase reagent was used to measure Renilla luciferase activity. Renilla luciferase fluorescence value was used as an internal reference. The activity ratio was used as the reporter gene activity value.

### Chromatin immunoprecipitation (ChIP)

Cells were cross-linked with 1% formaldehyde and shaken for 15 min. The cells were harvested and the nuclear chromatin was broken into 300 bp fragments using an ultrasonic disruptor. Incubate anti-HOXD10-A, anti-Snail1-(promoter region) (Purchased from Abcam, USA) in the diluted chromatin crushed product. After combination with protein A agarose beads (Millipore, USA), the ChIP product were measured using Chemiluminescence Imaging (Clinx Ltd., China).

### Statistical methods

The data were expressed by mean ± standard deviation and analyzed by SPSS16.0 software (IBM, USA). T-test was used ro compare two groups. Statistic difference between groups was analyzed using one-way analysis of variance. P < 0.05 was believed to be statistically significant differences.

### Ethics approval

The ethics committee of Fujian Medical University Union Hospital had reviewed and approved all experimental protocols. All patients had read and signed the informed consent.
